# A New General Hospital for Putney

**Published:** 1905-06-03

**Authors:** 


### A NEW GENERAL HOSPITAL FOR PUTNEY.

A MEETING of the inhabitants of Putney was held on May 25, under the presidency of Sir Arthur Jelf, to form a committee for the foundation of a hospital in the parish. The hospital is made possible by the generosity of the late Mr. Henry Chesters, a resident in Putney, who left the residue of his estate, estimated at £60,000, to the Haberdashers' Company, of which he was a member, for the purpose of using the income to maintain a general hospital there, on condition that such a hospital be built within twenty years of the testator's death. Failing this the money is to go to Guy's Hospital. An appeal was made for funds to build, in the first place, a hospital of twenty beds, but with the administrative portion sufficiently large to admit of the number of beds being increased to 150. A site of about an acre and a half has been secured on Putney Common, largely through the energy and generosity of Mr. W. J. Lancaster, the first Mayor of the Borough of Wandsworth, and promises of support have been received from many of the dwellers in the neighbourhood, including the Bishop of Rochester, who accompanied his donation by a very sympathetic letter.

The urgency of the need for a hospital in Wandsworth was brought out by Sir Henry Burdett in his paper on "Hospital Sites and Population in London" in March 1903. It was there shown that the south-western district, which includes Wandsworth and Battersea, had a ratio of total hospital beds to population of only one to 6,445 people. The ratio for the whole of the south-western division, which includes Battersea, was only one hospital bed to 6,074 people. The south-western district contains a population of upwards of 400,000 people, and only one general hospital, containing 30 beds, whilst the special hospital bed accommodation amounts to 36. We hope, therefore, that the seasonable generosity of the late Mr. Chesters, who may have been moved to make this legacy from reading the paper referred to, will receive the practically unanimous support of all classes of people in Wandsworth, and that it may so secure a general hospital of adequate proportions under the best auspices and management.

